# Unraveling the Efficacy of Therapeutic Interventions for Short Cervix: Insights from a Retrospective Study for Improved Clinical Management

**DOI:** 10.3390/medicina59061018

**Published:** 2023-05-24

**Authors:** Alina-Madalina Luca, Elena Bernad, Dragos Nemescu, Cristian Vaduva, Anamaria Harabor, Ana-Maria Adam, Valeriu Harabor, Aurel Nechita, Cristina Strobescu, Raluca Mogos, Alexandru Carauleanu, Ingrid-Andrada Vasilache, Demetra Socolov

**Affiliations:** 1 Department of Obstetrics and Gynecology, ‘Grigore T. Popa’ University of Medicine and Pharmacy, 700115 Iasi, Romania; 2 Department of Obstetrics and Gynecology, Faculty of Medicine, “Victor Babes” University of Medicine and Pharmacy, 300041 Timisoara, Romania; 3 Department of Mother and Child Medicine, Faculty of Medicine, University of Medicine and Pharmacy, 200349 Craiova, Romania; 4 Clinical and Surgical Department, Faculty of Medicine and Pharmacy, ‘Dunarea de Jos’ University, 800216 Galati, Romaniaadam.anamaria89@gmail.com (A.-M.A.);; 5 Department of Vascular Surgery, University of Medicine and Pharmacy “Grigore T. Popa”, 700111 Iasi, Romania

**Keywords:** preterm birth, progesterone, cervical cerclage, pessary

## Abstract

*Background and Objectives*: Preterm birth (PTB) is associated with important neonatal mortality and morbidity. The aim of this study was to retrospectively evaluate the average treatment effects on the treated and the efficacity of various therapeutic interventions for PTB in a cohort of patients with singleton pregnancies and short cervical lengths. *Materials and Methods*: This observational retrospective study included 1146 singleton pregnancies at risk of PTB that were segregated into the following groups: intravaginal progesterone (group 1), Arabin pessary (group 2), McDonald cerclage (group 3), intravaginal progesterone and Arabin pessary (group 4), and intravaginal progesterone and cerclage (group 5). Their treatment effects were evaluated and compared. *Results*: All evaluated therapeutic interventions significantly reduced the occurrence of late and early preterm births. The risk of late and early PTB was lowered for those pregnant patients who received progesterone and pessaries or progesterone and cerclage in comparison with those who received only progesterone. The extremely PTB risk of occurrence was significantly lowered only by the administration of progesterone in association with cervical cerclage in comparison with progesterone monotherapy. *Conclusions*: The combined therapeutic interventions had the highest efficacy in preventing preterm birth. An individualized evaluation is needed to establish the best therapeutic approach in particular cases.

## 1. Introduction

Preterm birth (PTB), defined as any birth before 37 complete weeks of gestation, is an important public health problem responsible for approximately 2.5 million neonatal deaths per year worldwide [1]. Gestational age sub-groups (such as extremely preterm, very preterm, moderate preterm, and late preterm), the occurrence of preterm birth (spontaneous versus medically induced), and pathophysiological background are examples of common classification criteria of categorization systems [2]. PTB may occur naturally, as a result of spontaneous preterm labor and/or preterm pre-labor membrane rupture, or under the direction of a healthcare professional by cesarean delivery or labor induction.

The complications of PTB include acute respiratory distress syndrome (ARDS), necrotizing enterocolitis (NE), sepsis, intraventricular hemorrhage (IVH), hypoxic–ischemic encephalopathy (HIE), seizures, and cerebral palsy, as well as feeding difficulties and visual or hearing impairment [3,4,5,6]. Follow-ups of these patients reveal a higher prevalence of neurodevelopmental problems, along with social-emotional and learning difficulties [7,8]. 

A plethora of risk factors for the prediction of preterm birth have been proposed [9,10,11,12], but only a few of them have remained consistent throughout the literature. Maternal characteristics are the most studied, and it was proven that ethnicity, extremes of maternal age, low maternal education, smoking, illicit drug consumption, personal history of preterm birth, short cervical length (less than 2.5 cm), and maternal comorbidities (vaginal or systemic infections, autoimmune disorders, thrombophilia, etc.) were significantly associated with the occurrence of PTB [13,14,15,16]. Moreover, some hormonal and vitamin imbalances have been proposed as risk factors for pregnancy complications, such as PTB [17]. Thus, correcting these imbalances will result in improving the overall health status of these patients [18,19]. 

Preterm birth has also been linked to placental, uterine, or fetal abnormalities such as placental abruption, placenta previa, polyhydramnios, uterine malformations, uterine fibromas, and fetal structural or chromosomal defects [20,21,22,23]. There is a lack of agreement over whether prior uterine surgery (curettage, hysteroscopy, myomectomy, and multiple previous cesarean surgeries) increases the risk of preterm birth or not, and although systematic reviews have found only modest associations, they were unable to account for all possible confounders [24,25,26,27].

Various treatment strategies for PTB have been proposed, including vaginal progesterone, pessaries, and cerclage, with or without the association of tocolysis. For a woman with a short cervix and a history of spontaneous preterm delivery, the National Institute for Health and Care Excellence’s (NICE) preterm birth guidelines suggest giving the option of vaginal progesterone or cervical cerclage [28]. NICE also advises women with low cervical lengths (25 m) or histories of spontaneous preterm birth to take into account vaginal progesterone [28]. Vaginal progesterone has recently been the subject of large, negative, randomized controlled trials [10,11], which have raised questions regarding its efficacy [29,30].

On the other hand, Care et al. evaluated, in a systematic review and meta-analysis, 61 trials that compared the efficacy of various interventions for the prevention of preterm birth in singleton pregnancies, and the authors concluded that vaginal progesterone was associated with fewer women with preterm births <34 weeks (odds ratio (OR): 0.50, 95% confidence interval (CI): 0.34–0.70), along with Shirodkar cerclage (effect size (ES): 0.06, 95% CI: 0.00–0.84), and vaginal pessary (ES: 0.65, 95% CI: 0.39 to 1.08) [31]. Still, there is a great heterogeneity regarding the recommendations of various therapeutic strategies, and the PTB prevention protocols differ between healthcare institutions. Moreover, current data from observational studies were determined after the evaluation of small cohorts of patients over short timeframes, thus providing low-quality evidence. 

In 2022, Pacagnella et al. published a multicenter, open-label, randomized controlled trial that evaluated the efficacy of the cervical pessary in addition to vaginal progesterone for the prevention of preterm birth in women with shortened cervixes, and the authors concluded that the combination therapy did not decrease rates of neonatal morbidity or mortality [32]. On the other hand, they showed that the combination progesterone–pessary had significantly lower rates of overall preterm births compared to monotherapy.

There are various formulations of progesterone that can be administered orally, intravaginally, or intramuscularly. A recent randomized clinical trial of 150 pregnant patients at risk of preterm birth, who had received oral Dydrogesterone (30 mg/day), 17α-hydroxyprogesterone caproate (17α-OHPC, 250 mg intramuscular, weekly), or nothing, showed that progesterone caproate obtained superior results in prolonging the latency period until birth and improving neonatal outcomes in comparison with oral progesterone and placebo [33].

Very few observational studies have evaluated the treatment effects of various strategies used in monotherapy or combined therapies. Therefore, the aim of this study was to retrospectively evaluate the average treatment effects and efficacies of various therapeutic interventions for preterm birth in a cohort of patients with singleton pregnancies and short cervical lengths. 

## 2. Materials and Methods

This observational retrospective study included 1146 singleton pregnancies with asymptomatic short cervixes that were evaluated at the tertiary maternity hospital ‘Cuza-Voda’, Iasi, Romania, between January 2017 and December 2021. Ethical approval for this study was obtained from the Institutional Ethics Committees of ‘Cuza-Voda’ Maternity Hospital (No. 2052/16.02.2021) and the University of Medicine and Pharmacy ‘Grigore T. Popa’ (No. 101/08.07.2021). Informed consent was waived for this study, but all participants included in the study signed a consent form for the use of anonymized clinical data in further studies. All methods were carried out in accordance with relevant guidelines and regulations. 

Inclusion criteria comprised singleton pregnancies with certain first-trimester dating, maternal age ≥18 years old, and short cervical lengths (less than 2.5 cm) that presented at our institution between 18 and 22 weeks of gestation for fetal morphological evaluation. The exclusion criteria referred to twin pregnancies, structural or chromosomal fetal abnormalities, patients with preterm labor, premature rupture of membranes, or vaginal infections, patients with clinical emergencies who could not receive one of the proposed therapeutic approaches, stillbirth, and incomplete medical records.

The risk of preterm birth was considered in the presence of short cervical length (less than 2.5 cm) measured by transvaginal ultrasound using an E8 scanner with a 5–15 MHz transvaginal probe (GE Medical Systems, Milwaukee, WI, USA), as recommended by ISUOG [34].

Each physician chose the therapeutic approach for these pregnant patients based on local protocols and international guidelines [35,36,37,38], while taking into account the patient’s preference and compliance with a specific treatment. Intravaginal progesterone was administered to asymptomatic patients with or without a personal history of PTB and short cervical length (less than 25 mm). The Arabin pessary was chosen for a patient with or without a personal history of PTB if the vaginal ultrasound indicated signs of cervical incompetence (cervical shortening and funneling). Cervical cerclage was recommended for a patient with a personal history of PTB and short cervical length or in the presence of major clinical modifications of the cervix (cervical effacement or dilation), with or without a protrusion of the amniotic sac, even in the absence of a personal history of PTB. Intravaginal progesterone was added to cervical cerclage or Arabin pessary at the physician’s discretion, especially when the cervical length was less than 15 mm.

The patients were segregated into the following groups depending on the employed therapeutic approach: intravaginal progesterone (200 mg/day)—group 1 (*n* = 562 patients), Arabin pessary—group 2 (*n* = 286 patients), McDonald cerclage—group 3 (*n* = 128 patients), intravaginal progesterone and Arabin pessary—group 4 (*n* = 101 patients), and intravaginal progesterone and cerclage—group 5 (*n* = 69 patients).

The evaluated outcomes were represented by preterm birth between 32 and 36 + 6 weeks of gestation (late preterm), 28 and 31 + 6 weeks of gestation (early preterm), and at less than 28 weeks of gestation (extremely preterm). From the patient’s medical records, we also retrieved demographic data, a personal history of preterm birth, thrombosis, or ischemic placental disease, and comorbidities (thrombophilia, autoimmune disorders, etc.), as well as neonatal outcomes, such as birth through cesarean delivery, Apgar scores at 1 and 5 min, neonatal intensive care unit admission (NICU), the presence of IVH, cerebral palsy, ARDS, necrotizing enterocolitis, the need for mechanical ventilation, and neonatal death.

Pearson’s chi-squared test was used to determine whether there is a statistically significant difference between the expected frequencies and the observed frequencies in one or more categories of clinical characteristics. For continuous variables, results were given as mean and standard deviation (SD), and between-group differences were assessed using ANOVA. For the multivariate analysis of treatment groups, we used multinomial logistic regression, adjusted for maternal age, smoking status, and the presence of comorbidities. For binary outcomes, relative risk (RR) and 95% CI values were calculated. We also calculated average treatment effects on the treated (ATT) using regression adjustment and compared the logarithmic odds ratios (logORs) of various therapeutic interventions for the evaluated outcomes. A *p*-value less than 0.05 was considered statistically significant. The statistical analyses were performed using STATA SE (version 17, 2022; StataCorp LLC, College Station, TX, USA).

## 3. Results

This observational retrospective study included 1146 pregnant patients with singleton pregnancies, segregated into five groups: intravaginal progesterone—group 1 (*n* = 562 patients), Arabin pessary—group 2 (*n* = 286 patients), McDonald cerclage—group 3 (*n* = 128 patients), intravaginal progesterone and Arabin pessary—group 4 (*n* = 101 patients), and intravaginal progesterone and cerclage—group 5 (*n* = 69 patients). 

The clinical characteristics of the evaluated groups and the results from the univariate analysis are presented in Table 1. Pregnant patients who underwent cervical cerclage had the highest rates of preterm births in their personal history (39.06%), followed by patients who received intravaginal progesterone and Arabin pessaries (30.69%) and intravaginal progesterone and cerclage (26.08%). We found a statistically significant difference regarding this aspect between groups (*p* < 0.001).

We evaluated the average treatment effects of various therapeutic interventions, and we described them considering the main outcomes. The average treatment effects on the treated (ATT) analysis (Table 2) revealed that all therapeutic interventions significantly reduced the occurrence of late and early preterm births. Progesterone in combination with cervical cerclage had the highest impact on the occurrence of both late (ATT = −0.28; 95%CI: −0.48–0.08; *p* = 0.006) and early (ATT = −0.21; 95%CI: −0.35–0.37; *p* = 0.009) PTB. On the other hand, only progesterone intravaginally administered significantly reduced the occurrence of extremely preterm birth (ATT = −0.07; 95%CI: −0.13–0.10; *p* < 0.001). 

**Figure 1:**
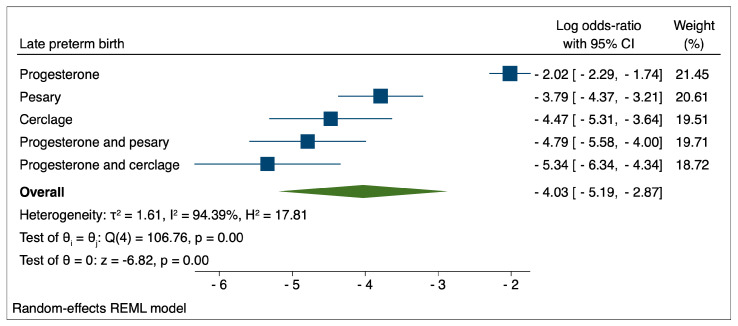
Comparison between therapeutic interventions for the reduction of late preterm birth.

**Figure 2:**
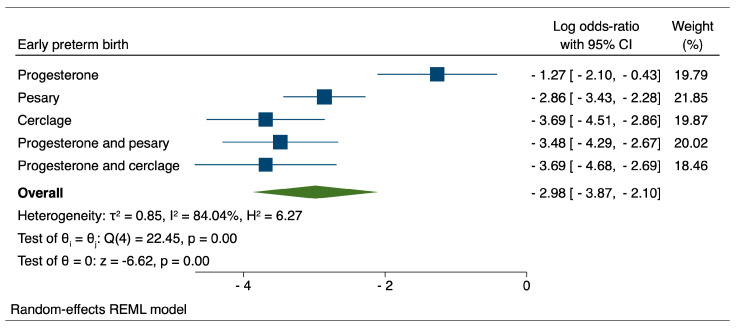
Comparison between therapeutic interventions for the reduction of early preterm birth.

**Figure 3:**
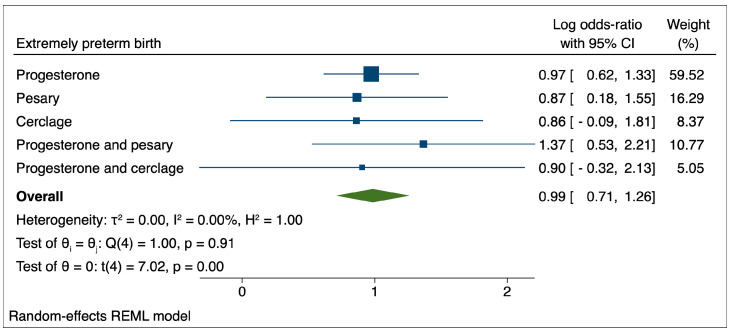
Comparison between therapeutic interventions for the reduction of extremely preterm birth.

Graphical representations of the comparisons between evaluated therapeutic interventions for the reduction of various types of preterm birth are presented in Figure 1, Figure 2 and Figure 3. The highest performance in the reduction of late PTB was achieved by the combination of progesterone and cerclage (logOR: −5.34; 95% CI: −6.34–−4.34), followed by progesterone and pessary (logOR: −4.79; 95% CI: −5.58–−4.00). The lowest performance, in this case, was achieved by the administration of progesterone in monotherapy (logOR: −2.02; 95% CI: −2.29–−1.74).

When evaluating the reduction of early preterm birth through therapeutic interventions, our results indicated that both cervical cerclage (logOR: −3.69; 95% CI: −4.51–−2.86) and the combination of progesterone and cerclage (logOR: −3.69; 95% CI: −4.68–−2.69) achieved similar performances, closely followed by the combination of progesterone and pessary (logOR: −3.48; 95%CI: −4.29–−2.67).

In the case of extremely preterm birth, the results indicated a non-significant influence of the evaluated therapeutic interventions over the pregnancy’s course and a tendency for these interventions to be associated with increased odds of PTB.

In our cohort of patients, the risk of occurrence of late preterm birth was significantly higher for pregnant patients who received progesterone in comparison with those who received Arabin pessaries (RR: 3.13; 95% CI: 2.42–4.04; *p* < 0.001) or cervical cerclage (RR: 2.73; 95% CI: 1.93–3.86; *p* < 0.001) (Table 3). On the other hand, the risk was significantly lower for patients who received progesterone and pessaries (RR: 0.36; 95% CI: 0.24–0.54; *p* < 0.001) or progesterone and cerclage (RR: 0.30; 95% CI: 0.18–0.52; *p* < 0.001) in comparison with those who received only progesterone, translating into a risk reduction of 64% for the first treatment option and 70% for the second treatment option. 

The risk of occurrence of early preterm birth was similarly increased in patients who received progesterone in comparison with those who received Arabin pessaries (RR: 3.73; 95% CI: 2.37–5.88; *p* < 0.001) or cervical cerclage (RR: 3.57; 95% CI: 1.87–6.83; *p* < 0.001). The risk was also lowered for those patients who received progesterone and pessaries (RR: 0.44; 95% CI: 0.24–0.78; *p* = 0.005) or progesterone and cerclage (RR: 0.49; 95% CI: 0.26–0.91; *p* ≤ 0.02) in comparison with those who received only progesterone, translating into a risk reduction of 56% for the first treatment option and 51% for the second treatment option. 

The same pattern of increased risk of extremely preterm birth was observed for patients who received progesterone in comparison with those who received Arabin pessaries (RR: 4.81; 95% CI: 2.49–9.26; *p* < 0.001) or cervical cerclage (RR: 4.16; 95% CI: 1.71–10.12; *p* = 0.001). However, the risk of occurrence was significantly lowered only by the administration of progesterone in association with cervical cerclage in comparison with progesterone monotherapy (RR: 0.27; 95% CI: 0.08–0.84; *p* = 0.02).

Finally, we evaluated and compared neonatal outcomes using multinomial logistic regression (Table 4). Our analysis revealed that late preterm neonates were born significantly more frequently through cesarean section (*p* < 0.001), required significantly more invasive ventilation (*p* < 0.001), and developed ARDS after birth (*p* < 0.001). Moreover, the Apgar scores at 1 and 5 min of less than seven were significantly prevalent in this group. 

Early preterm neonates were admitted to the NICU significantly more frequently (*p* < 0.001), were diagnosed more frequently with necrotizing enterocolitis and ARDS (*p* < 0.001), and required more invasive ventilation (*p* < 0.001). In addition, they were more prone to receive an Apgar score of less than seven at 1 (*p* = 0.007) and 5 min (*p* < 0.001) and had higher rates of neonatal death (*p* < 0.001).

Extremely preterm neonates were the most fragile group, having significantly higher rates of NICU admission (*p* < 0.001), necrotizing enterocolitis (*p* < 0.001), intraventricular hemorrhage (*p* < 0.001), cerebral palsy (*p* = 0.035), visual or hearing impairment (*p* = 0.003), neonatal deaths (*p* < 0.001), ARDS (*p* < 0.001), and invasive ventilation (*p* < 0.001), as well as lower Apgar scores at 1 (*p* = 0.008) and 5 min (*p* = 0.03).

## 4. Discussion

This retrospective study evaluated the effectiveness of various therapeutic interventions for preterm birth and compared their treatment effects, considering preterm delivery as an outcome in three gestational age categories. The average treatment effects on the treated indicated that all therapeutic interventions significantly reduced the occurrence of late and early preterm birth, with the highest impact achieved using the combination of progesterone with cervical cerclage. On the other hand, only progesterone intravaginally administered significantly reduced the occurrence of extremely preterm birth.

Similar results were obtained when we compared the occurrence of various types of preterm birth depending on the therapeutic interventions employed. The highest performance for the reduction in late PTB was achieved using the combination of progesterone and cerclage, followed by progesterone and pessary. The lowest performance, in this case, was achieved by the administration of progesterone in monotherapy.

When evaluating the reduction in early preterm birth using therapeutic interventions, our results indicated that both cervical cerclage and the combination of progesterone and cerclage achieved similar performances, closely followed by the combination of progesterone and pessary. On the other hand, in the case of extremely preterm birth, the results indicated a non-significant influence of the evaluated therapeutic interventions over the pregnancy’s course and a tendency for these interventions to be associated with increased odds of PTB.

These results can be explained by the fact that extremely preterm labor is more difficult to manage and that therapeutic interventions such as cervical cerclage are often performed in emergencies. A meta-analysis of 12 observational studies, which evaluated the effectiveness of emergency cerclage versus expectant management on maternal and perinatal outcomes, indicated that cerclage was superior to expectant management for the reduction in preterm delivery rates before 28 and 32 weeks of gestation, but these results were based on low-quality evidence [39]. Nevertheless, the intraoperative rupture of membranes is a risk associated with emergency cerclage that ranges from 4% to 9%, and this procedure’s apparently positive effects appear to be greatly reduced in the presence of chorioamnionitis [40,41,42]. 

In our cohort of patients, the risk of the occurrence of late preterm birth was significantly higher for pregnant patients who received progesterone in comparison with those who received Arabin pessaries or cervical cerclage. On the other hand, the risk was significantly lower for patients who received progesterone and pessaries or progesterone and cerclage in comparison with those who received only progesterone. Our results are in line with previously published data.

A Cochrane systematic review that evaluated the efficacy of cervical pessaries for preventing preterm birth in comparison with other therapeutic interventions in women with singleton pregnancies at risk of preterm delivery indicated that the cervical pessary reduced the risk of delivery before 34 weeks (RR: 0.72; 95%CI: 0.52–1.02) or before 37 weeks (RR: 0.89; 95%CI: 0.73–1.09) in comparison with vaginal progesterone administration [43].

Another Cochrane systematic review that evaluated the effect of cervical cerclage versus other therapeutic interventions in patients at risk of premature delivery concluded that there is not enough quality evidence to determine whether cerclage is more or less effective than progesterone administration, either vaginally or intramuscularly, for the prevention of PTB [44]. On the contrary, a recent indirect comparison meta-analysis concluded that both vaginal progesterone and cervical cerclage are equally effective in preventing PTB [45].

Our results indicated that the risk of early PTB was also lowered for those pregnant patients who received progesterone and pessaries or progesterone and cerclage in comparison with those who received only progesterone, translating into a risk reduction of 56% for the first treatment option, and 51% for the second treatment option. Our results were confirmed by a randomized controlled trial that evaluated the outcomes of combined therapy (cervical cerclage with progesterone) in comparison with progesterone monotherapy and that outlined pregnancy prolongation for preterm labor at 24–28 weeks in the case of combined intervention [46]. Moreover, another recent randomized controlled trial concluded that the cervical pessary was not non-inferior to vaginal progesterone for preventing spontaneous birth before 34 weeks of gestation in pregnant women with short cervixes [47].

The extremely PTB risk of occurrence was significantly lowered only by the administration of progesterone in association with cervical cerclage in comparison with progesterone monotherapy. Similar results were obtained in a retrospective cohort study by Enakpene et al., that revealed a higher performance of the combination therapy (cerclage and progesterone) in preventing extremely preterm birth (<28 weeks of gestation) in comparison with progesterone monotherapy (RR: 0.23; 95%CI: 0.10−0.54, *p* = 0.001) [48].

Our study also outlined significant personal histories of preterm birth for pregnant patients who received cervical cerclage, intravaginal progesterone and Arabin pessary, or intravaginal progesterone and cerclage, in accordance with the published data [49]. Regarding neonatal outcomes, both late and early preterm neonates were significantly associated with adverse outcomes, such as acute respiratory distress syndrome, the need for mechanical ventilation, or low Apgar scores. Extremely preterm neonates were significantly more fragile, with higher rates of neonatal deaths, intraventricular hemorrhage, cerebral palsy, and visual or hearing impairment in addition to the previously mentioned adverse neonatal outcomes, which are commonly encountered after preterm deliveries as stated in the literature [50,51,52].

The limitations of this study are represented by its retrospective approach, unicentric design, and small sample size. On the other hand, this study has the advantage of following pregnancy outcomes in a 4-year timeframe for patients with singleton pregnancies at risk of PTB who received therapeutic interventions in monotherapy or combined therapies. 

Further prospective multicentric randomized controlled trials should be conducted in order to comparatively evaluate the performance of combined therapeutic interventions such as cerclage and progesterone versus progesterone monotherapy for the prevention of PTB.

## 5. Conclusions

All evaluated therapeutic interventions significantly reduced the occurrence of late and early preterm births. The highest performance in the reduction of late PTB was achieved by the combination of progesterone and cerclage, followed by progesterone and pessary. Both cervical cerclage and the combination of progesterone and cerclage achieved similar performances regarding the rates of early PTB, closely followed by the combination of progesterone and pessaries. 

The risk of late and early PTB was lowered for those pregnant patients who received progesterone and pessaries or progesterone and cerclage in comparison with those who received only progesterone. The extremely PTB risk of occurrence was significantly lowered only with the administration of progesterone in association with cervical cerclage in comparison with progesterone monotherapy.

Further prospective studies will be needed in order to elucidate the performance of combined therapies for the prevention of PTB. In addition, an individualized assessment of pregnant patients, with the identification of maternal or sonographic risk factors for preterm birth, will allow prompt administration of treatment tailored to their risk profile. 

## Figures and Tables

**Table 1:** Univariate analysis of the clinical characteristics of the patients included in our study.

Patient’s Data	Group 1 (*n =* 562 Patients)	Group 2 (*n =* 286 Patients)	Group 3 (*n =* 128 Patients)	Group 4 (*n =* 101 Patients)	Group 5 (*n =* 69 Patients)	*p*-Value
Maternal age, years (mean and standard deviation)	29.46 ± 6.59	30.34 ± 6.54	29.98 ± 6.60	29.83 ± 6.85	30.67 ± 6.19	0.31
Medium (*n*/%)	Rural = 291 (51.9%) Urban = 270 (48.1%)	Rural = 138 (48.1%) Urban = 149 (51.9%)	Rural = 68 (53.1%) Urban = 60 (46.9%)	Rural = 52 (51.5%) Urban = 49 (48.5%)	Rural = 42 (60.9%) Urban = 39.1 (47%)	0.41
Smoking (*n*/%)	Yes = 13 (2.3%)	Yes = 10 (3.49%)	Yes = 7 (5.5%)	Yes = 5 (5.0%)	Yes = 4 (5.8%)	0.19
Personal history of preterm birth (*n*/%)	Yes = 34 (6.04%)	Yes = 47 (16.43%)	Yes = 50 (39.06%)	Yes = 31 (30.69%)	Yes = 18 (26.08%)	<0.001
Personal history of thrombosis (*n*/%)	Yes = 2 (0.35%)	Yes = 1 (0.34%)	Yes = 0 (0%)	Yes = 0 (0%)	Yes = 0 (0%)	0.47
Diabetes (*n*/%)	Yes = 6 (1.1%)	Yes = 2 (0.7%)	Yes = 1 (0.8%)	Yes = 1 (1%)	Yes = 0 (0%)	0.91
Thrombophilia (*n*/%)	Yes = 5 (0.88%)	Yes = 2 (0.69%)	Yes = 1 (0.8%)	Yes = 0 (0%)	Yes = 0 (0%)	0.98
Personal history of autoimmune disorders (*n*/%)	Yes = 18 (3.2%)	Yes = 14 (4.89%)	Yes = 8 (6.25%)	Yes = 5 (4.95%)	Yes = 0 (0%)	0.33
Personal history of ischemic placental disease (*n*/%)	Yes = 7 (1.24%)	Yes = 4 (1.39%)	Yes = 2 (1.56%)	Yes = 1 (1%)	Yes = 0 (0%)	0.98
Cervical length, mm (mean and standard deviation)	22.1 ± 2.16	20.25 ± 2.21	19.75 ± 1.70	18.75 ± 3.59	16.5 ± 3.87	0.14

**Table 2:** Average treatment effects on the treated of the evaluated therapeutic interventions for preterm birth.

Treatment	Late Preterm Birth				Early Preterm Birth				Extremely Preterm Birth			
	ATT	95%CI Lower Bound	95%CI Upper Bound	*p*-Value	ATT	95%CI Lower Bound	95%CI Upper Bound	*p*-Value	ATT	95%CI Lower Bound	95%CI Upper Bound	*p*-Value
Progesterone	−0.14	−0.19	−0.09	<0.001	−0.07	−0.13	0.11	<0.001	−0.07	−0.13	0.10	<0.001
Pessary	−0.16	−0.28	−0.05	<0.001	−0.10	−0.17	0.21	0.03	0.06	−0.01	0.13	0.09
Cerclage	−0.14	−0.31	−0.01	0.01	−0.08	−0.19	0.23	0.02	0.06	−0.04	0.17	0.255
Progesterone and pessary	−0.18	−0.35	−0.01	0.03	−0.07	−0.21	0.16	0.01	0.16	−0.03	0.35	0.09
Progesterone and cerclage	−0.28	−0.48	−0.08	0.006	−0.21	−0.35	0.37	0.009	0.09	−0.09	0.29	0.306

Table legend: ATT—average treatment effect on the treated; CI—confidence interval.

**Table 3:** Calculated relative risks for various therapeutic interventions in relationship with the evaluated outcomes.

Therapeutic Intervention	Late Preterm Birth		Early Preterm Birth		Extremely Preterm Birth	
	RR and 95% CI	*p*-Value	RR and 95% CI	*p*-Value	RR and 95% CI	*p*-Value
Progesterone vs. pessary	3.13 (2.42–4.04)	<0.001	3.73 (2.37–5.88)	<0.001	4.81 (2.49–9.26)	<0.001
Progesterone vs cerclage	2.73 (1.93–3.86)	<0.001	3.57 (1.87–6.83)	<0.001	4.16 (1.71–10.12)	0.001
Cerclage vs. pessary	0.87 (0.57–1.32)	0.52	1.04 (0.49–2.20)	0.11	1.15 (0.40–3.28)	0.78
Progesterone + pessary vs progesterone alone	0.36 (0.24–0.54)	<0.001	0.44 (0.24–0.78)	0.005	0.59 (0.28–1.23)	0.15
Progesterone + pessary vs. cerclage	0.79 (0.47–1.32)	0.37	1.58 (0.69–3.61)	0.27	2.45 (0.82–7.35)	0.10
Progesterone + pessary vs. pessary	1.14 (0.72–1.82)	0.55	1.65 (0.82–3.28)	0.15	2.83 (1.13–7.11)	0.05
Progesterone + cerclage vs. progesterone alone	0.30 (0.18–0.52)	<0.001	0.49 (0.26–0.91)	0.02	0.27 (0.08–0.84)	0.02
Progesterone + cerclage vs. pessary	0.96 (0.53–1.73)	0.91	1.84 (0.88–3.81)	0.10	1.31 (0.37–4.60)	0.66
Progesterone + cerclage vs. cerclage	0.84 (0.45–1.58)	0.60	1.76 (0.74–4.17)	0.19	1.13 (0.28–4.56)	0.85
Progesterone + pessary vs. progesterone +cerclage	1.18 (0.61–2.29)	0.61	0.89 (0.39–2.01)	0.79	1.61 (0.43–5.92)	0.47

Table legend: RR—relative risk; CI—confidence interval; vs.—versus.

**Table 4:** Pregnancy and neonatal outcomes in preterm deliveries.

Outcome	Late Preterm Birth		Early Preterm Birth		Extremely Preterm Birth	
	aOR and 95% CI	*p*-Value	aOR and 95% CI	*p*-Value	aOR and 95% CI	*p*-Value
Cesarean delivery	2.11 (0.35–5.41)	<0.001	0.95 (0.70–1.29)	0.78	0.88 (0.49–1.57)	0.68
Apgar score at 1 min < 7	1.82 (1.42–2.31)	<0.001	1.79 (0.68–4.41)	0.007	1.83 (0.19–4.51)	0.008
Apgar score at 5 min < 7	1. 21 (0.45–3.58)	<0.001	2.65 (1.55–4.55)	<0.001	1.28 (0.45–3.44)	0.03
NICU admission	0.96 (0.67–1.38)	0.84	1.26 (0.17–4.21)	<0.001	0.72 (0.18–2.87)	<0.001
Necrotizing enterocolitis	0.44 (0.16–1.85)	0.79	1.04 (0.55–2.65)	<0.001	1.97 (0.49–3.35)	<0.001
Invasive ventilation	1.39 (0.54–3.09)	<0.001	1.42 (0.73–2.10)	<0.001	1.47 (0.25–2.75)	<0.001
ARDS	1.56 (0.37–3.32)	<0.001	1.38 (0.38–2.44)	<0.001	2.32 (0.92–3.37)	<0.001
Cerebral palsy	0.96 (0.08–4.83)	0.67	0.20 (−0.30–0.72)	0.42	1.99 (0.26–4.04)	0.035
Visual or hearing impairment	0.48 (0.02–2.52)	0.43	0.90 (−0.86–2.68)	0.31	1.67 (0.08–4.68)	0.003
Intraventricular hemorrhage	0.56 (0.37–1.32)	0.06	0.38 (−0.38–1.14)	0.32	2.32 (0.92–3.37)	<0.001
Neonatal death	0.68 (0.36–1.82)	0.79	1.04 (−0.55–2.65)	<0.001	1.97 (0.49–3.35)	<0.001

Table legend: aOR—adjusted OR; CI—confidence interval; NICU—neonatal intensive care unit; ARDS—acute respiratory distress syndrome.

## Data Availability

The data presented in this study are available upon request from the corresponding author. The data are not publicly available because of local policies.
